# Supplementary material for: Gene networks and pathways for plasma lipid traits via multitissue multiomics systems analysis
Source: J Lipid Res. 2021 Jan 5;62:100019. doi: 10.1194/jlr.RA120000713 (PMC7873371; doi:10.1194/jlr.RA120000713)
Supplement: Figure S3 [file mmc7.pdf]

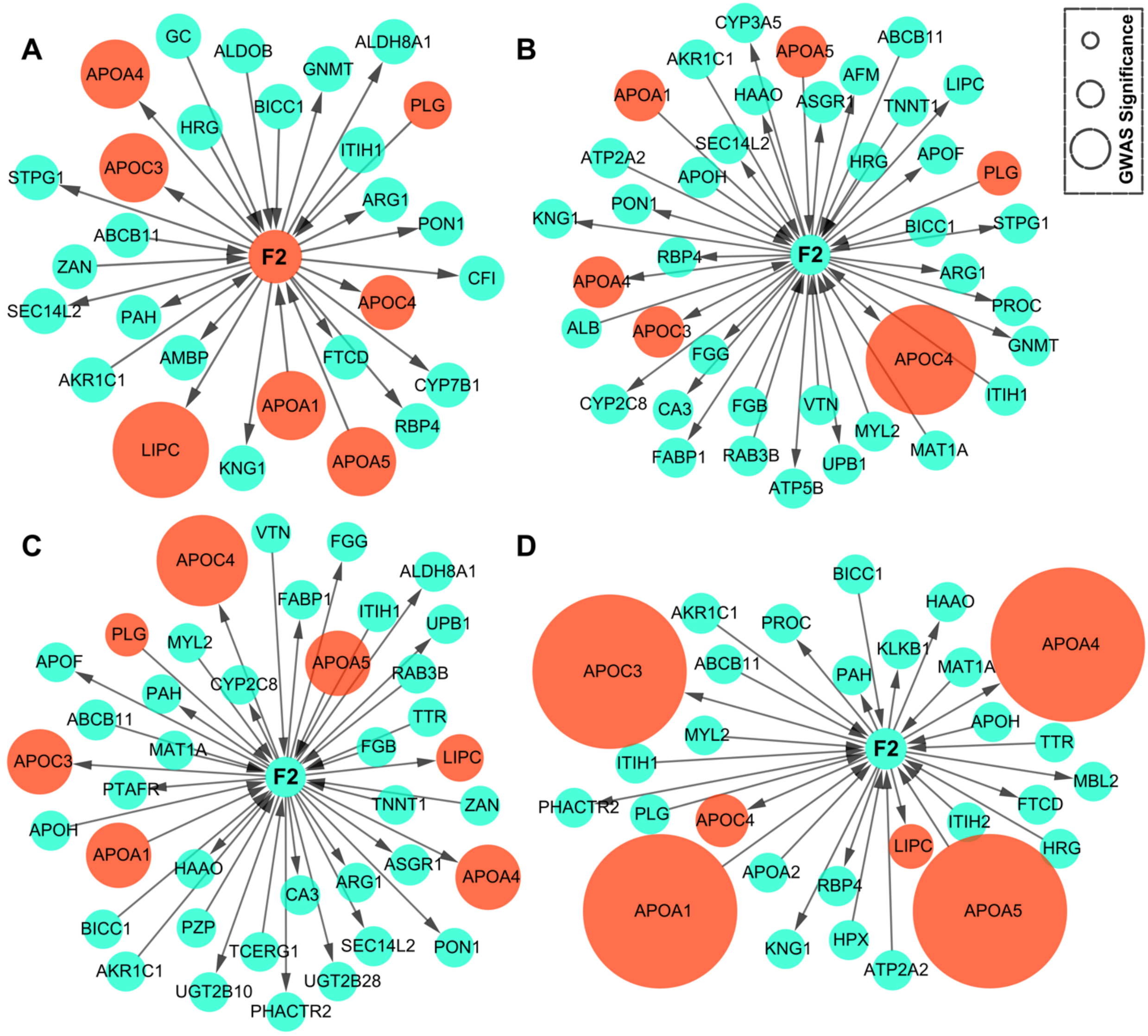

Supplemental Figure S3. GWAS genes in Neighboring genes of Gene *F2* in human Bayesian networks. Panel **(A-D)** represent GWAS susceptibility genes around gene *F2* for HDL, LDL, TC, and TG respectively. The interactions come from a combined Bayesian network from different human tissues, including adipose, liver, blood, kidney, muscle, and brain. The node size corresponds to the GWAS significance.
