# Supplementary material for: Gene networks and pathways for plasma lipid traits via multitissue multiomics systems analysis
Source: J Lipid Res. 2021 Jan 5;62:100019. doi: 10.1194/jlr.RA120000713 (PMC7873371; doi:10.1194/jlr.RA120000713)
Supplement: Table S1 [file mmc9.pdf]

Supplemental Table S1. Studies included in the positive control datasets for lipid traits.

Traits [a]	PubMed IDs	References	Reported Genes
TC	20686565	[15]	CELSR2, PSRC1, SORT1, APOE, APOC1, APOC2, LDLR, APOB, APOA1, APOC3, APOA4, APOA5, HMGCR, ABCG5, ABCG8, ANGPTL3, DOCK7, CSPG3, CILP2, PBX4, TRIB1, TIMD4, HAVCR1, ABCA1, GCKR, HP, HPR, DHX38, PCSK9, PPP1R3B, FADS1, FADS2, FADS3, ABO, LIPC, LIPG, HLA, TOP1, LPA, HNF1A, IRF2BP2, TOMM20, CETP, MOSC1, HNF4A, CYP7A1, BRAP, ST3GAL4, NPC1L1, TMEM57, LDLRAP1, C6orf106, MAFB, UBASH3B, FRK, FUT2, FLJ36070, GPAM, ERGIC3, DNAH11, PLEC1, NAT2, IDOL, TTC39B, RAF1, OSBPL7, RAB3GAP1, TRPS1, HFE, HIST1H4C, SPTY2D1, GVI1, EVI5, NCAN, TOMM40
	20370913	[16]	
	20066028	[17]	
	19060911	[18]	
TG	22171074	[19]	APOA5, ALDH2, GCKR, TBL2, MLXIPL, LPL, ZNF259, APOA1, APOC3, APOA4, BUD13, APOB, DOCK7, ANGPTL3, BAZ1B, BCL7B, TRIB1, CILP2, ZNF101, AFF1, C5orf35, FADS1, APOE, APOC1, APOC2, CSPG3, PBX4, FADS2, FADS3, PLTP, HLA, GALNT2, NAT2, LIPC, CETP, JMJD1C, TIMD4, HAVCR1, KLHL8, FRMD5, ANKRD55, MAP3K1, COBLL1, LRP1, TYW1B, CCDC92, ZNF664, PINX1, XKR6, CAPN3, IRS1, CYP26A1, MSL2L1, CTF1, PLA2G6, DSCAML1, TOMM40, NCAN, AMAC1L2, ATG4C, LOC440069, MGC13125, C8orf35, SLC18A1, KIAA0999, LOC645044
	21909109	[20]	
	20864672	[21]	
	20686565	[15]	
	19074352	[22]	
	19060911	[18]	
	19060906	[23]	
	18193044	[24]	
	18193046	[25]	
	18193043	[26]	
	17463246	[27]	
LDL	23067351	[28]	APOB, MYLIP, GMPR, PCSK9, CELSR2, HMGCR, TRIB1, BUD13, ZNF259, APOA5, APOA4, APOC3, APOA1, LDLR, SF4, CILP2, APOE, APOC1, APOC4, APOC2, PPP1R3B, PSRC1, SORT1, ABCG5, ABCG8,
	21977987	[29]	
	21059979	[30]	

	20864672	[21]	HP, HPR, DHX38, TIMD4, HAVCR1, CSPG3, PBX4, ABO, FADS1, FADS2, FADS3, TOP1, ANGPTL3, DOCK7, LPA, HNF1A, ST3GAL4, HLA, PLEC1, CETP, IRF2BP2, TOMM20, IDOL, NPC1L1, CBLN3, KIAA0323, MOSC1, TMEM57, LDLRAP1, HFE, HIST1H4C, DNAH11, BRAP, GPAM, FRK, CYP7A1, OSBPL7, MAFB, NCAN, TOMM40, CR1L, AR, B3GALT4, GCKR
	20686565	[15]	
	19060911	[18]	
	19060906	[23]	
	19060910	[31]	
	18802019	[32]	
	18262040	[33]	
	18193044	[24]	
	18193043	[26]	
	18179892	[34]	
HDL	23505323	[35]	SLC12A3, NLRC5, HERPUD1, CETP, TSPAN16, SPC24, RAB3D, LOC55908, KANK2, DOCK6, PPP1R3B, NIPSNAP3A, NIPSNAP3B, ABCA1, MYL2, C12orf51, OAS3, LPL, PVRL2, TOMM40, APOE, FADS1, TTC39B, GALNT2, APOB, ZNF259, APOA5, APOA4, APOC3, APOA1, MYO1H, KCTD10, UBE3B, MMAB, MVK, LIPC, GFOD2, LCAT, LIPG, FADS2, FADS3, PLTP, APOC1, APOC2, TRIB1, LRP4, NR1H3, LILRA3, LILRB2, HNF4A, KLF14, SCARB1, STARD3, CMIP, TRPS1, SLC39A8, ABCA8, COBLL1, CCDC92, ZNF664, ZNF648, MACF1, PABPC4, MLXIPL, IRS1, C6orf106, PGS1, RPS3A, MC4R, SBNO1, LACTB, UBE2L3, LRP1, CITED2, UBASH3B, LPA, ANGPTL4, PDE3A, ADM, AMPD3, ARL15, NUP93, MADD, FOLH1, CTCF, PRMT8, ACAA2
	21909109	[20]	
	22303337	[36]	
	20864672	[21]	
	20686565	[15]	
	19359809	[37]	
	20031564	[38]	
	19060911	[18]	
	19060906	[23]	
	19060910	[31]	
	20031538	[39]	
	18193044	[24]	
	18193043	[26]	

Traits [a]: TG: Triglycerides; LDL: Low-Density Lipoprotein Cholesterol; HDL: High-Density Lipoprotein Cholesterol.
