# Supplementary material for: Gene networks and pathways for plasma lipid traits via multitissue multiomics systems analysis
Source: J Lipid Res. 2021 Jan 5;62:100019. doi: 10.1194/jlr.RA120000713 (PMC7873371; doi:10.1194/jlr.RA120000713)
Supplement: Table S2 [file mmc10.pdf]

Supplemental Table S2. Studies that produced the gene co-expression modules and Bayesian network models of gene-gene interactions.

Tissues	Data sources	References
Human gene expression resources and gene interaction networks		
Aortic endothelial cells	149 heart transplant donors	[40]
Adipose tissue and blood	1,675 individuals from two Icelandic cohorts	[7]
Blood	1,469 unrelated individuals	[8]
Fibroblasts, lymphoblastoid cells and T cells	Umbilical cords of 85 Western European individuals	[41]
Liver	427 individuals	[10]
Liver and adipose tissue	1,008 obese patients	[9]
Lymphoblastoid cells	400 children of families with a proband with asthma	[42]
	60 HapMap participants of European descent	[43]
	270 HapMap participants	[44]
	726 HapMap3 participants	[45]
	30 European and 30 Yoruba HapMap participants	[46]
Lymphoblastoid cells, skin and adipose tissue	150 female twins	[14]
Monocytes	1,490 unrelated individuals	[47]
Mouse gene expression resources and gene interaction networks		
Liver, adipose tissue, kidney, heart, muscle, brain	C57BL/6J x A/J cross	[6]
Liver, adipose tissue, muscle, brain	C57BL/6J x C3H ApoE -/-	[11,12]
Liver, adipose tissue, muscle	C57BL/6J x C3H wildtype	[10]
Islet cells, adipose tissue, liver, muscle, hypothalamus	C57BL/6J x BTBR Lep^{ob}	[13]
