# Supplementary material for: Gene networks and pathways for plasma lipid traits via multitissue multiomics systems analysis
Source: J Lipid Res. 2021 Jan 5;62:100019. doi: 10.1194/jlr.RA120000713 (PMC7873371; doi:10.1194/jlr.RA120000713)
Supplement: Table S3 [file mmc11.pdf]

Supplemental Table S3. Primer pairs for RT qPCR

Genes	*Forward (5'-3')*	*Reverse (5'-3')*
F2	*TTCCCGAACCAGATATGAGC*	*CTGTCTGCTTGTCTGGCAAA*
F2 siRNA-1	*GGAACAGCUUACCAGCCAATT*	*UUGGCUGGUAAGCUGUUCCTT*
F2 siRNA-2 (chosen)	*GCGAUAUCGCUCUGCUCAATT*	*UUGAGCAGAGCGAUAUCGCTT*
F2 siRNA-3	*GCUCCGGGCUGGUUAUAAATT*	*UUUAUAACCAGCCCGGAGCTT*
Sc siRNA	*UUCUCCGAACGUGUCACGUTT*	*ACGUGACACGUUCGGAGAATT*
Abcb11	*GGACAATGATGTGCTTGTGG*	*CACACAAAGCCCCTACCAGT*
Apoa4	*GTCACCTTCCAACGTGGAGT*	*GATGTTTCCCGGAGTCAGAA*
Apoa5	*GAGTCGAGTGCTGCACCATA*	*ACGTGTGAGTTTGTGGGACA*
Apof	*GAAAGGATGCCACCACAGAT*	*CCACCCATCCATCCATCTAC*
Fabp1	*GGAAGGACATCAAGGGGGTG*	*TCACCTTCCAGCTTGACGAC*
Gc	*ACTCAGAGTCCCCTGCTGAA*	*GGTGTTGGGTGTTTTTGTCC*
Hrg	*ATGGTCCACCACATGGACAC*	*AGTGGAGGGAGTCGGTAGAC*
Lipc	*GACTGGATCTCCCTGGCATA*	*AGGTGAACTTTGCTCCGAGA*
Plg	*GCTGCCTGTGATTGAGAACA*	*CTCGAAGCAAACCAGAGGTC*
Proc	*GGACCTGGACATCAAGGAGA*	*GCAGATGGGCACTATGGTTT*
Snrbp2	*AAGAGATCCCTGTATGCCCTT*	*GTGGATGAACCCAGTTCCTTAAA*
Gpt	*TCCAGGCTTCAAGGAATGGAC*	*CAAGGCACGTTGCACGATG*
Itga6	*TGCAGAGGGCGAACAGAAC*	*GCACACGTCACCACTTTGC*
Spry44	*GCAGCGTCCCTGTGAATCC*	*TCTGGTCAATGGGTAAGATGGT*
Lep	*GAGACCCCTGTGTCGGTTC*	*CTGCGTGTGTGAAATGTCATTG*
Pparg	*CCATTCTGGCCCACCAAC*	*AATGCGAGTGGTCTTCCATCA*
Cebpa	*GCGGGCAAAGCCAAGAA*	*GCGTTCCCGCCGTACC*
Srebp1	*CTCAGCAGCCACCATCTAGCCT*	*GCTGATGCCTGCAGTCTTCACG*
Fasn	*CTG AGATCCCAGCACTTCTTGA*	*GCCTCCGAAGCCAAATGAG*
Adipoq	*GATGGCACTCCTGGAGAGAA*	*TCTCCAGGCTCTCCTTTCCT*
Lipe	*ACAGTGCAGGTGGGAATCTC*	*GCCTAGTGCCTTCTGGTCT*
Cd36	*GCAGGTCTATCTACGCTGTG*	*GGTTGTCTGGATTCTGGAGG*
Fabp4	*TGAAATCACCGCAGACGACA*	*ATAACACATTCCACCACCAGC*
Beta actin	*GCAGGAGTACGATGAGTCCG*	*ACGCAGCTCAGTAACAGTCC*
